# Novel conformation-specific monoclonal antibodies against amyloidogenic forms of transthyretin

**DOI:** 10.1186/1750-1172-10-S1-P1

**Published:** 2015-11-02

**Authors:** Jeffrey N Higaki, Avi Chakrabartty, Natalie J Galant, Bradley Hammerson, Ronald Torres, Joshua Salmans, Robin Barbour, Gene G Kinney

**Affiliations:** 1 Prothena Biosciences Inc, N/A, 94080, South San Francisco, USA; 2 University of Toronto, Department of Medical Biophysics, M5G 2C1, Toronto, Canada; 3 University of Toronto, Princess Margaret Cancer Centre, University Health Network, Department of Medical Biophysics, M5G 2C1, Toronto, Canada

## Background

Amyloidoses are a progressive, systemic disease caused by the accumulation in tissues of misfolded proteins that induces multiorgan dysfunction. The most common hereditary form is transthyretin amyloidosis (ATTR), caused by the accumulation of transthyretin protein (TTR). There are no approved pharmacologic therapies for ATTR in the United States, and liver transplantation is the only disease-modifying treatment. TTR, a redundant thyroxin transport protein, comprises four single-chain monomers assembled into a tetrameric complex in its native state. During the amyloidogenic process, the tetramer dissociates into monomeric subunits that then undergo conformational change, making them more prone to aggregation and fibril formation. Comparison of the crystal structure of tetrameric TTR and the monomeric TTR identified a region that is inaccessible in the tetramer but exposed upon monomer dissociation. By targeting this site with a monoclonal antibody (mAb), it might be possible to prevent TTR monomers from assembling into fibrils without influencing the function of the native tetramer. The objective of this study was to produce mAbs targeting this exposed epitope of monomeric TTR and to (a) demonstrate conformational specificity toward misfolded versus native forms of TTR and (b) determine whether they are able to recognize TTR deposits in diseased tissue.

## Methods

Mice were immunized with a multiple antigenic peptide comprising the target sequence identified in the structural analysis of TTR. Clones were screened for reactivity against misfolded TTR fibrils and counter-screened against native tetrameric TTR. Selected mAbs were characterized by sandwich ELISA, SPR, and Western blot. Immunohistochemistry was performed in combination with Congo red and thioflavin-T staining to demonstrate specificity to TTR-amyloid in ATTR patient-derived tissue sections.

## Results

Four mAbs were identified that bind to the target epitope on monomeric and nonnative conformations of TTR. These mAbs bound nonnative forms of TTR (KD values 7.7-18.6 nM) but, importantly, did not recognize native tetrameric TTR. These mAbs also recognized TTR deposits in a variety of ATTR heart tissues. They did not recognize control heart tissue (normal or AL amyloidosis) or the native tetrameric TTR present in human liver tissue.

## Conclusions

Conformation-specific mAbs immunoreact with an amyloidogenic epitope of TTR but not with native tetrameric TTR. These mAbs specifically recognize TTR deposits in ATTR heart tissue, not in control tissue (normal and AL amyloidosis). These novel mAbs may be useful in preventing deposition and/or enhancing clearance of TTR amyloid in ATTR patients.

